# Exploring the Legal Implications of Benign Prostatic Hyperplasia Surgeries in the United States: A Comprehensive Analysis of Two Decades of Lawsuits

**DOI:** 10.7759/cureus.39335

**Published:** 2023-05-22

**Authors:** Joao Porto, Maria Camila Suarez Arbelaez, Mohamadhusni Zarli, Mariam Ahumada, Robin C Schard, Timothy Loftus, Sanjaya Swain, Robert Marcovich, Hemendra N Shah

**Affiliations:** 1 Desai Sethi Urology Institute, University of Miami, Miami, USA; 2 Dr. Kiran C. Patel College of Osteopathic Medicine, Nova Southeastern University, Fort Lauderdale, USA; 3 School of Law, University of Miami, Miami, USA; 4 Department of Public Health Sciences, School of Medicine, University of Miami, Miami, USA

**Keywords:** transurethral resection of prostate, urinary incontinence, litigation, lawsuit, surgery, benign prostatic hyperplasia

## Abstract

Introduction and objective: The United States (US) currently faces a medical malpractice crisis, and a survey done in 2006 informed that 63% of urologists faced an average of 2.1 medical malpractice lawsuits. Surgery for benign prostatic hyperplasia (BPH) is performed by 95% of US urologists. Hence, we postulated that these procedures might be responsible for a substantial number of medical malpractice lawsuits. We examined claims related to BPH surgery in various US courts.

Materials and methods: Data were collected from Westlaw and LexisNexis databases using the keywords "benign prostatic hyperplasia," "enlarged prostate," "surgery," and "malpractice" to search for cases from the entire US from January 2000 to December 2021. We extracted details such as the type of procedure, the plaintiff and defendant, the nature of the allegation, the alleged complications, the verdict, and the compensation amount.

Results: We found 30 cases in which the most common procedure was transurethral resection of the prostate (37%), with inadequate postoperative care as the most frequent reason for claims (33%). Urologists were the most frequently processed professionals (57%). The postsurgical outcomes that resulted in lawsuits were urinary incontinence (23%), erectile dysfunction (13%), and urinary retention (13%). Interestingly, 43% of the patients were inmates. Plaintiffs won only two (7%) cases: colon perforation after interstitial laser coagulation with Indigo laser and recto-urethral fistula after transurethral microwave therapy.

Conclusion: Most lawsuits were related to postoperative incontinence and erectile dysfunction, with the verdict favoring the defendant in most cases. Inmates were the plaintiffs in a relatively high percentage of cases. Only two cases resulted in a plaintiff victory, wherein both cases presented unexpected and serious postsurgical complications.

## Introduction

Medical malpractice has become a growing concern in the United States (US) in recent decades [1]. Although malpractice lawsuits were intended to address lapses in patient safety, they have contributed significantly to yearly healthcare expenditures. A study by the Harvard School of Public Health revealed that medical malpractice accounts for over $55 billion in annual healthcare costs, making it a considerable burden on the healthcare system [2]. Specialties with high liability are particularly susceptible to facing medical malpractice lawsuits [3]. After analysis of claims data from 1985 to 2007, urology was ranked 12th out of 28 medical specialties in terms of both the frequency of medical malpractice lawsuits and the amount of compensation awarded to plaintiffs [4,5]. Additionally, according to a recent survey of physicians conducted in 2021, urology is the fourth most sued medical specialty, behind plastic surgery, general surgery, and orthopedics [6]. Moreover, it is estimated that a urologist will experience an average of 2.1 malpractice lawsuits in their career [7].

According to a report by the Physician Insurance Association of America, 162 malpractice claims (MCs) against urologists were closed in 2003. Of these claims, 20.4% resulted in payment to the patient, with the total indemnity amounting to $13,652,620 and an average indemnity of $413,716. The median indemnity was $270,000 and the largest indemnity paid in a particular case was $1,426,814. These statistics highlight the significant financial impact of medical malpractice lawsuits in the field of urology and underline the need for measures to reduce the risk of such lawsuits and improve patient safety [8].

The current literature on litigation review for benign prostatic hyperplasia (BPH) surgeries is limited to transurethral procedures in a single legal database, i.e., "Westlaw" (Thomson Reuters, Eagan, MN) [9,10]. This approach may overlook non-transurethral procedures such as open, laparoscopic, and robotic procedures. To address this limitation, we conjectured that combining multiple legal databases, specifically Westlaw and LexisNexis (Relx Inc., New York, NY), would yield a more comprehensive collection of relevant cases due to the fact that only 7% of searched legal cases can be found in different databases, while 40% of cases are exclusive of only one database [11,12]. Furthermore, we speculated that the number of lawsuits related to BPH surgeries has increased over the past two decades, possibly due to changes in medical practices, patient expectations, and the introduction of new minimally invasive surgical therapies (MIST).

To test these hypotheses, we analyzed lawsuits related to all BPH surgeries over the last 20 years to identify the most reported symptoms, focusing on the most common procedures, reasons for the claims, and involved professionals. We also examined the legal outcomes of these lawsuits and the verdicts in favor of either the plaintiff or defendant. Understanding the factors that contributed to these lawsuits and the legal outcomes that resulted from them is crucial to reducing the number of MCs and improving patient care.

## Materials and methods

We retrospectively reviewed two primary online legal databases for both state and federal statutes, Westlaw and LexisNexis, by searching targeted keywords. Specifically, we included the terms "benign prostatic hyperplasia," "enlarged prostate," "surgery," and "malpractice." Natural language algorithms for both databases appear to be based on different criteria, thereby giving different results. The search period was from January 1st, 2000, to December 31st, 2021. The data encompassed legal cases from across the US and we identified 506 legal cases using these search terms. To ensure the integrity of our analysis, we took steps to exclude legal processes that were not directly related to the surgical procedure under investigation, as well as duplicate cases in the databases. After a thorough review process, we selected 27 reports that satisfied our inclusion criteria for the study. However, there were two cases where multiple patients underwent surgery by the same surgeon and jointly decided to sue the doctor. In one of the reports, two patients filed a joint lawsuit together against the doctor, and in the other report, three patients did the same. To ensure fairness and avoid any potential bias toward individual defendants, we treated each plaintiff as a separate case and included each as a unique case in our study. As a result, a total of 30 cases were included in the analysis. Information about each plaintiff was collected and analyzed in the subsequent stages of the study. In each of these cases, we meticulously extracted a range of information that was pertinent to our research question. Specifically, we recorded details related to the type of surgical procedure, the location of the case (including city and state), the parties involved in the case (both plaintiff and defendant), the nature of the allegations being made, any reported complications that occurred, the clinical outcome of the procedure, the verdict that was reached, and the amount of compensation that was awarded. Furthermore, we conducted an analysis of the average population size from 2000 to 2020 in each state that was involved in a lawsuit related to BPH to calculate the average population per lawsuit [13].

## Results

**Figure 1:**
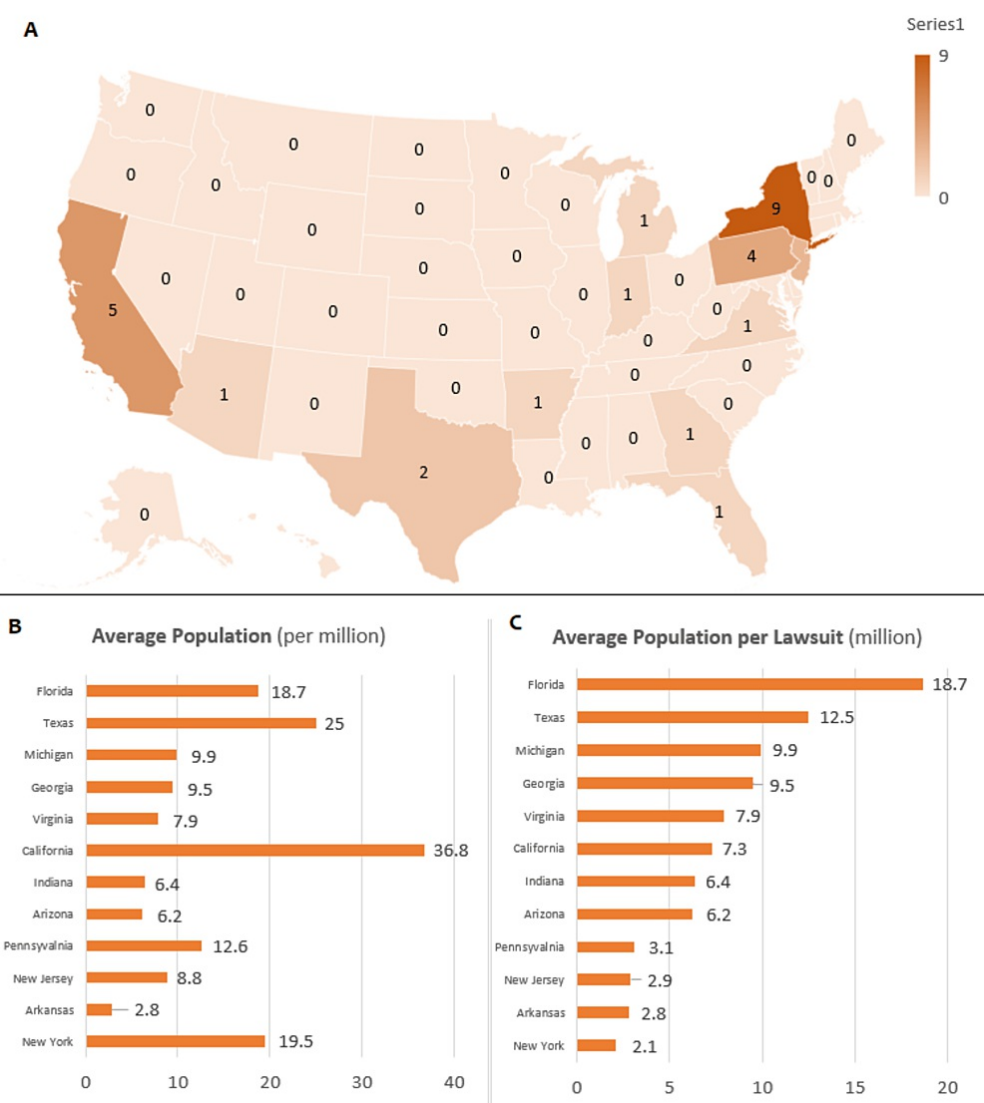
(A) Number of lawsuits for benign prostatic hyperplasia surgery per state from 2000 to 2021. (B) The average population per state from 2000 to 2021 (million). (C) The average population per lawsuit from 2000 to 2021 (million).

Our study analyzed a total of 30 cases from different regions of the country, with the highest number of reports coming from New York (30%), California (17%), and Pennsylvania (13%) (Figure 1A). Among states that faced lawsuits related to BPH procedures, New York had the lowest average population per lawsuit, while Florida had the highest (Figures 1B, 1C). Of 30 cases, 17 were reported in Westlaw and 13 additional cases were identified in the LexisNexis database. The procedures most associated with litigation were transurethral resection of the prostate (TURP; 37%), transurethral microwave therapy (TUMT; 10%), and photo-vaporization of the prostate (PVP; 10%) (Table 1). Among the allegations made by the plaintiffs, the most common was inadequate postoperative care, which accounted for 33% of the claims. Other allegations included the procedure being performed by an inappropriate physician (20%), improper surgical technique (20%), and failure to obtain appropriate informed consent (17%). While various medical professionals were involved in the cases, urologists were the most common defendants, accounting for 57% of the cases (Table 2). In terms of clinical outcomes, urinary incontinence was the most frequent cause of litigation, representing 23% of the cases. Erectile dysfunction and urinary retention followed closely behind, each 13%. Interestingly, a considerable proportion of the patients were inmates, accounting for 43% of the cases. Of these, 92% claimed a violation of the Eighth Amendment of the US Constitution, alleging that their rights to receive proper care were denied. Ultimately, the plaintiffs were only successful in achieving a favorable verdict in two cases, representing a mere 7% of the total lawsuits analyzed. These cases involved rare complications following MIST, such as colon perforation after interstitial laser coagulation (ILC) with the Indigo system and recto-urethral fistula after TUMT.

**Table 1:** Summary of BPH surgery lawsuits. BPH: benign prostatic hyperplasia; HoLEP: holmium laser enucleation of the prostate; ILC: interstitial laser coagulation; LUTS: lower urinary tract symptoms; PVP: photo-vaporization of the prostate; TUMT: transurethral microwave therapy; TUNA: transurethral needle ablation; TURP: transurethral resection of the prostate; UTI: urinary tract infection; WIT: water-induced thermotherapy.

Procedure	Nature of allegation	Clinical outcome	Final verdict
HoLEP	Inadequate informed consent	Erectile dysfunction	Defendant
ILC	Negligence in products liability	Colon perforation	Plaintiff
PVP	Inadequate preoperative care	Urinary incontinence	Unknown
PVP	Improper surgical technique	Urinary incontinence	Unknown
PVP	Misdiagnosis	Death	Unknown
TUMT	Improper surgical technique	Rectal-urethral fistula	Plaintiff
TUMT	Unnecessary medical treatment	Urinary incontinence	Defendant
TUMT	Procedure not performed by an appropriate physician	Urinary retention	Defendant
TUNA	Inadequate postoperative care	Urinary retention	Defendant
TUNA	Misdiagnosis	LUTS	Defendant
TURP	Improper surgical technique	Urinary incontinence	Defendant
TURP	Inadequate informed consent	Erectile dysfunction	Defendant
TURP	Procedure not performed by an appropriate physician	UTI	Defendant
TURP	Procedure not performed by an appropriate physician	UTI	Defendant
TURP	Inadequate postoperative care	Urinary incontinence	Defendant
TURP	Inadequate postoperative care	Urinary retention	Defendant
TURP	Procedure not performed by an appropriate physician	Required operative intervention	Defendant
TURP	Procedure not performed by an appropriate physician	LUTS	Defendant
TURP	Procedure not performed by an appropriate physician	LUTS	Defendant
TURP	Inadequate postoperative care	Erectile dysfunction	Defendant
TURP	Inadequate postoperative care	UTI	Defendant
WIT	Improper surgical technique	Stricture	Defendant
Unknown	Inadequate informed consent	Urinary incontinence	Defendant
Unknown	Improper surgical technique	Erectile dysfunction	Defendant
Unknown	Inadequate postoperative care	LUTS	Defendant
Unknown	Inadequate postoperative care	Retrograde ejaculation	Defendant
Unknown	Failure to arrange follow up	Unknown	Defendant
Unknown	Improper surgical technique	Urinary incontinence	Defendant
Unknown	Inadequate postoperative care	Pain	Defendant
Unknown	Inadequate postoperative care	Pressure ulcer	Defendant

**Table 2:** Main characteristic of lawsuits filed against benign prostatic hyperplasia procedures.

Total No.	30
Plaintiff (%)	
Patient/patient and spouse	26 (87%)
Patient's estate	4 (13%)
Defendants, multiple providers named per case (%)	
Urologist	17 (57%)
Emergency physician	4 (13%)
Physician's assistant	3 (10%)
Other non-urological providers	9 (30%)
Hospital sued (%)	12 (40%)
Prison system sued (%)	5 (17%)
Alleged malpractice (reasons)	
Failure to provide adequate informed consent	5
Inappropriate preoperative care	1
Improper surgical technique	6
Misdiagnosis	2
Inappropriate postoperative care	10
Failure to arrange follow-up	4
Negligence in products liability	1
Unnecessary medical treatment	1
Procedure not performed by an appropriate physician	6
Total available awards data	$3.9 million

## Discussion

Although insurance database review indicates that BPH procedures are the second most prevalent cause of MC in the field of urology, just behind kidney surgeries, only 30 cases of litigation were reported from both state and federal courts over the last two decades in two legal databases from the US. This discrepancy noted in the number of MCs clearly indicates that most MCs are settled out of court, and few claims are filed in state or federal courts [14]. However, when relatively rare complications occur, plaintiffs may have a greater chance of success in their lawsuits.

Historically, TURP is the gold-standard surgical treatment for BPH, and its complications accounted for 37% of lawsuits in our study [15,16]. We noted that urinary incontinence was the most common reason for allegations, followed by sexual dysfunction (13%). This can be explained by the fact that 95% and 92% of patients with BPH considered erectile and ejaculatory function, respectively, to be important factors when choosing a surgical procedure [17]. Our findings revealed that while common complications such as urinary incontinence and erectile dysfunction may be a reason for litigation, these alleged complications typically do not result in a verdict in favor of the plaintiff. Hence appropriate preoperative counseling of the patient and documentation of the same should reduce the risk of MC related to this known complication of TURP as reported earlier by other authors [9,14,18].

Surprisingly, complications associated with MIST were found to be associated with negative outcomes for the defendant. TUMT was argued to result in a urethral-rectal fistula due to improper technique, which led to a lawsuit with a final verdict in favor of the plaintiff with a value of $3.9 million [19]. In the past, the Food and Drug Administration (FDA) issued a warning that the use of TUMT devices was linked to thermal injuries that could lead to rectal fistula [20]. Another case with a negative verdict for the defendant involved ILC, a procedure that provides low-temperature coagulation and is controlled by a thermometer within the device. We identified a lawsuit where a patient had a colon perforation in which a probe was inadvertently passed through the prostate and into the colon. An extensive discussion related to the doctors’ responsibility was conducted and the patient also sued the company for negligence related to product liability. Previous studies related to this procedure reported no major complications [21-24].

Patients may initiate MC due to inappropriate postoperative care, as seen in 33% of allegations in our study. Similarly, 43% of urology-related allegations in England over a 20-year period were also linked to postoperative care [25]. However, Benson and coauthors' earlier investigation into urology-related litigation in the US revealed that improper performance (36%), misdiagnosis (15%), and failure to supervise a case (5.6%) were the most common reasons for indemnity [5]. We found that most cases were filed in New York, California, and Pennsylvania (Figure 1). Notably, we identified two groups of individuals who sued a New York-based urologist for issues related to residents. These individuals claimed that the procedures were performed by a resident while the provider was absent from the operating room. Golan et al. demonstrated that 12.5% of suits involving urology trainees are related to the failure of supervision [26]. However, this situation had a verdict in favor of the defendant.

Our study revealed a significant finding related to the incarcerated population as noted previously [9]. Thirteen individuals filed lawsuits against the correctional system, non-urological healthcare providers, and urologists. Of these, 12 plaintiffs (92%) alleged a violation of the Eight Amendment, which states, “Excessive bail shall not be required, nor excessive fines imposed, nor cruel and unusual punishments inflicted.” This constitutional provision prohibits the imposition of excessive fines or bail and the infliction of any form of cruel and unusual punishment. In the context of health care, this amendment has been interpreted to mean that prisoners have a right to receive adequate medical care [27]. Ganem et al. conducted a study that included a subset of the population who were prisoners (30%), and among this group, they found that there were no verdicts in their favor [9]. After examining these processes, we discovered that they were frequently inadequately written, displaying insufficient information about the procedures conducted (with 75% of cases lacking a defined procedure), and without details about the reasons that prompted legal action. Overall, there is a lack of research on medical malpractice lawsuits for inmates across different medical fields, not just urology. It is likely that the lack of patient autonomy, distrust in the healthcare system, and limited access to resources are key drivers of legal action. Further research is needed to better understand the underlying causes and implications of MC in the inmate population and to identify strategies for improving the quality and safety of healthcare delivery in prisons. Healthcare providers working with prisoners should be proactive in providing comprehensive education and addressing any potential concerns.

Although we assessed two of the largest legal research databases in the US, investigating medical malpractice can be challenging due to the decentralized nature of the legal system, as well as the fact that many cases are either settled or dismissed [14]. Additionally, the databases may not include data from smaller courts or jurisdictions, and there may be a time lag between the occurrence of a legal case and its inclusion in the databases. Thus, it is important to acknowledge that not all cases are included in these databases. Some lawsuits were missing information about types of BPH surgery and were also missing key information, such as the final verdict and number of awards. Only one case in our study had publicly available information on the amount of the award [19]. Despite these limitations, we believe that our findings can help urologists and other healthcare providers understand the causes of MC and take appropriate precautions to prevent lawsuits. In most cases, lawsuits can be prevented through better pre-surgical counseling and physicians managing patients' expectations more effectively. The need to maintain careful documentation throughout the patient care process cannot be underestimated, in addition to the importance of providing special attention to patients during the postoperative period when they may feel more vulnerable. Furthermore, MIST procedures for BPH are advancing rapidly and although considered minimally invasive and safe, can remotely lead to severe complications that could become ground for an MC. It is essential to provide patients with comprehensive information about all potential future complications. Additionally, ongoing research is necessary to identify areas of patient dissatisfaction that could be appropriately addressed with the goal of minimizing the risk of MC.

## Conclusions

This study has revealed that verdicts in most claims related to the inherent and expected risks of medical procedures resulted in favor of the defendants. Nevertheless, rare and severe adverse events particularly after MIST procedures were responsible for the verdict in favor of the plaintiffs. There was an increase in MCs filed by prisoners alleging a violation of the Eight Amendment, although all verdicts returned in favor of the defendants.
