# Correction: Exploring the Legal Implications of Benign Prostatic Hyperplasia Surgeries in the United States: A Comprehensive Analysis of Two Decades of Lawsuits

**DOI:** 10.7759/cureus.c157

**Published:** 2024-01-31

**Authors:** Joao Porto, Maria Camila Suarez Arbelaez, Mohamadhusni Zarli, Mariam Ahumada, Robin C Schard, Timothy Loftus, Sanjaya Swain, Robert Marcovich, Hemendra N Shah

**Affiliations:** 1 Desai Sethi Urology Institute, University of Miami, Miami, USA; 2 Dr. Kiran C. Patel College of Osteopathic Medicine, Nova Southeastern University, Fort Lauderdale, USA; 3 School of Law, University of Miami, Miami, USA; 4 Department of Public Health Sciences, School of Medicine, University of Miami, Miami, USA

This article has been corrected at the request of the authors to add two authors, Ms. Robin C. Schard and Dr. Timothy Loftus, who were mistakenly omitted from the original author list. All authors have agreed to the revised author list. In addition, Ms. Robin C. Schard and Dr. Timothy Loftus have been removed from the Acknowledgements section since they are now listed as authors.

